# Production of highly efficient cellulase mixtures by genetically exploiting the potentials of *Trichoderma reesei* endogenous cellulases for hydrolysis of corncob residues

**DOI:** 10.1186/s12934-017-0825-3

**Published:** 2017-11-21

**Authors:** Yuanchao Qian, Lixia Zhong, Jia Gao, Ningning Sun, Yifan Wang, Guoyong Sun, Yinbo Qu, Yaohua Zhong

**Affiliations:** 1 State Key Laboratory of Microbial Technology, School of Life Sciences, Shandong University, Jinan, 250100 People’s Republic of China; 2 Shandong Institute for Food and Drug Control, Jinan, 250101 People’s Republic of China; 3 Anaesthesiology Department of the Second Hospital of Shandong University, Jinan, 250100 People’s Republic of China

**Keywords:** *Trichderma reesei*, CBH2, EG2, BGL1, Genetic engineering, Optimization, Saccharification

## Abstract

**Background:**

*Trichoderma reesei* is one of the most important fungi utilized for cellulase production. However, its cellulase system has been proven to be present in suboptimal ratio for deconstruction of lignocellulosic substrates. Although previous enzymatic optimization studies have acquired different types of in vitro synthetic mixtures for efficient lignocellulose hydrolysis, production of in vivo optimized cellulase mixtures by industrial strains remains one of the obstacles to reduce enzyme cost in the biofuels production from lignocellulosic biomass.

**Results:**

In this study, we used a systematic genetic strategy based on the *pyrG* marker to overexpress the major cellulase components in a hypercellulolytic *T. reesei* strain and produce the highly efficient cellulase mixture for saccharification of corncob residues. We found that overexpression of CBH2 exhibited a 32-fold increase in the transcription level and a comparable protein level to CBH1, the most abundant secreted protein in *T. reesei*, but did not contribute much to the cellulolytic ability. However, when EG2 was overexpressed with a 46-fold increase in the transcription level and a comparable protein level to CBH2, the engineered strain QPE36 showed a 1.5-fold enhancement in the total cellulase activity (up to 5.8 U/mL FPA) and a significant promotion of saccharification efficiency towards differently pretreated corncob residues. To assist the following genetic manipulations, the marker *pyrG* was successfully excised by homologous recombination based on resistance to 5-FOA. Furthermore, BGL1 was overexpressed in the EG2 overexpression strain QE51 (*pyrG*-excised) and a 11.6-fold increase in BGL activity was obtained. The EG2–BGL1 double overexpression strain QEB4 displayed a remarkable enhancement of cellulolytic ability on pretreated corncob residues. Especially, a nearly complete cellulose conversion (94.2%) was found for the delignified corncob residues after 48 h enzymatic saccharification.

**Conclusions:**

These results demonstrate that genetically exploiting the potentials of *T. reesei* endogenous cellulases to produce highly efficient cellulase mixtures is a powerful strategy to promote the saccharification efficiency, which will eventually facilitate cost reduction for lignocellulose-based biofuels.

## Background

Depletion of fossil fuels and deterioration of ecological environments have attracted intensive attentions toward the utilization of renewable lignocellulosic biomass for production of biofuels, such as cellulosic ethanol [1, 2]. An essential step in conversion of the lignocellulosic materials to ethanol or other liquid fuels is the enzymatic hydrolysis of polysaccharides into fermentable sugars [3]. However, the cost of cellulolytic enzymes remains one of the major hurdles to the development of a viable lignocellulosic ethanol industry [4, 5]. One promising approach to address this problem is design of more efficient, and thus cheaper, enzyme systems to promote commercialization of the bioconversion processes.

It is well-known that a multitude of enzymatic activities are required to degrade cellulose, the most abundant constituent of lignocellulosic biomass, into glucose as a fermentable sugar for ethanol production [4, 5]. These enzymes include cellobiohydrolases (CBHs, exactly CBH1 and CBH2), endoglucanases (EGs), and β-glucosidases (BGLs), which act in concert to hydrolyze cellulose [4, 5]. CBH1 and CBH2 move processively along cellulose chains cleaving off cellobiose units from the reducing ends and non-reducing ends, respectively, while EGs hydrolyze internal glycosidic bonds randomly within the chain and BGLs ultimately convert the oligosaccharides into glucose [6–8]. The complementary activities among the individual enzymes are considered to be responsible for synergistic effects, whereby the cellulase mixture can exhibit substantially higher activity than the sum of the component enzymes [9, 10]. Thus, the efficiency of the cellulolytic enzyme system depends not only on properties of individual enzymes but also their ratio in the multienzyme cocktail [4]. Optimization of the cellulase mixture by altering their ratio has become an important strategy for enzyme improvement [11, 12]. The reconstituted cellulase mixtures based on combining purified major component enzymes have been shown to perform as well as or even surpass the performance of commercial cellulases in hydrolysis of various cellulosic substrates [13–15]. For example, the in vitro design of minimal enzyme mixtures with only three major cellulases (CBH1, CBH2 and EG1) could reach 80.0% of the cellulose hydrolysis yield obtained with a commercial enzyme preparation [14]. However, the preparation of individual enzymes can still be tedious and relatively expensive, thus hindering the industrial application of the in vitro optimized cellulase mixture for lignocellulose bioconversion.

Current commercial cellulase preparations are mainly derived from the filamentous fungus *Trichoderma reesei*, which secretes all the core enzymes essential to the complete hydrolysis of lignocellulose [16]. The secreted cellulases contain two CBHs (CBH1 and CBH2) and at least four EGs (EG1, EG2, EG3 and EG5) that act in a synergistic manner to degrade the cellulosic materials, together with BGL1 and related hemicellulases [17–19]. CBH1 and CBH2 are the major cellulase components, which account for 50–60 and 10–15% of the total secreted protein by *T. reesei*, respectively [14]. Nonetheless, the specific activity of CBH2 is approximately twice that of CBH1 toward crystalline cellulose [20]. Further data displayed that a maximum CBH1–CBH2 synergism should reach a molar ratio of around 2:1 [12]. EG1 and EG2 are the two main EG activities, and their protein levels together constitute 6–20% of the total protein secreted [21]. Efforts to determine the EG activity have shown that EG2 has approximate twofold the specific activity of EG1 and accounts for most of the EG activity [22, 23]. In addition to the CBH and EG activities, low levels of BGL activity have long been considered as the major drawback, leading to incomplete conversion of cellobiose to glucose in the cellulose hydrolysis process [24]. In consequence, the respective activities in the *T. reesei* cellulase system appear to be present in suboptimal ratios for lignocellulose degradation. Intensive research efforts with genetic engineering strategies have been done to increase single cellulase components in *T. reesei* for strain improvement [25–28]. However, the cellulolytic potential of the endogenous cellulase system is still not sufficiently exploited, since multiple genetic manipulations in *T. reesei* have not been applied to optimizing the enzyme cocktail.

In our previous work, overexpression of BGL1 in *T. reesei* exhibited a 17.1-fold increase in BGL activity and provided much better performance on the enzymatic saccharification efficiency [26]. Here, we adopted a systematic genetic strategy based on the *pyrG* marker to overexpress the major cellulase components in a hypercellulolytic *T. reesei* strain. Individual overexpression of CBH2 or EG2 was firstly conducted and compared for cellulase production as well as saccharification efficiency. To assist multiple genetic manipulations, the marker *pyrG* was removed by homologous recombination based on resistance to 5-FOA. Furthermore, BGL1 was overexpressed in the EG2 overexpression strain to optimize the cellulase system. Differently pretreated corncob residues were finally used as substrates to assess the saccharification efficiency of the enzyme complexes.

## Results

### Overexpression of the native *cbh2* in *T. reesei* QP4

**Fig. 1:**
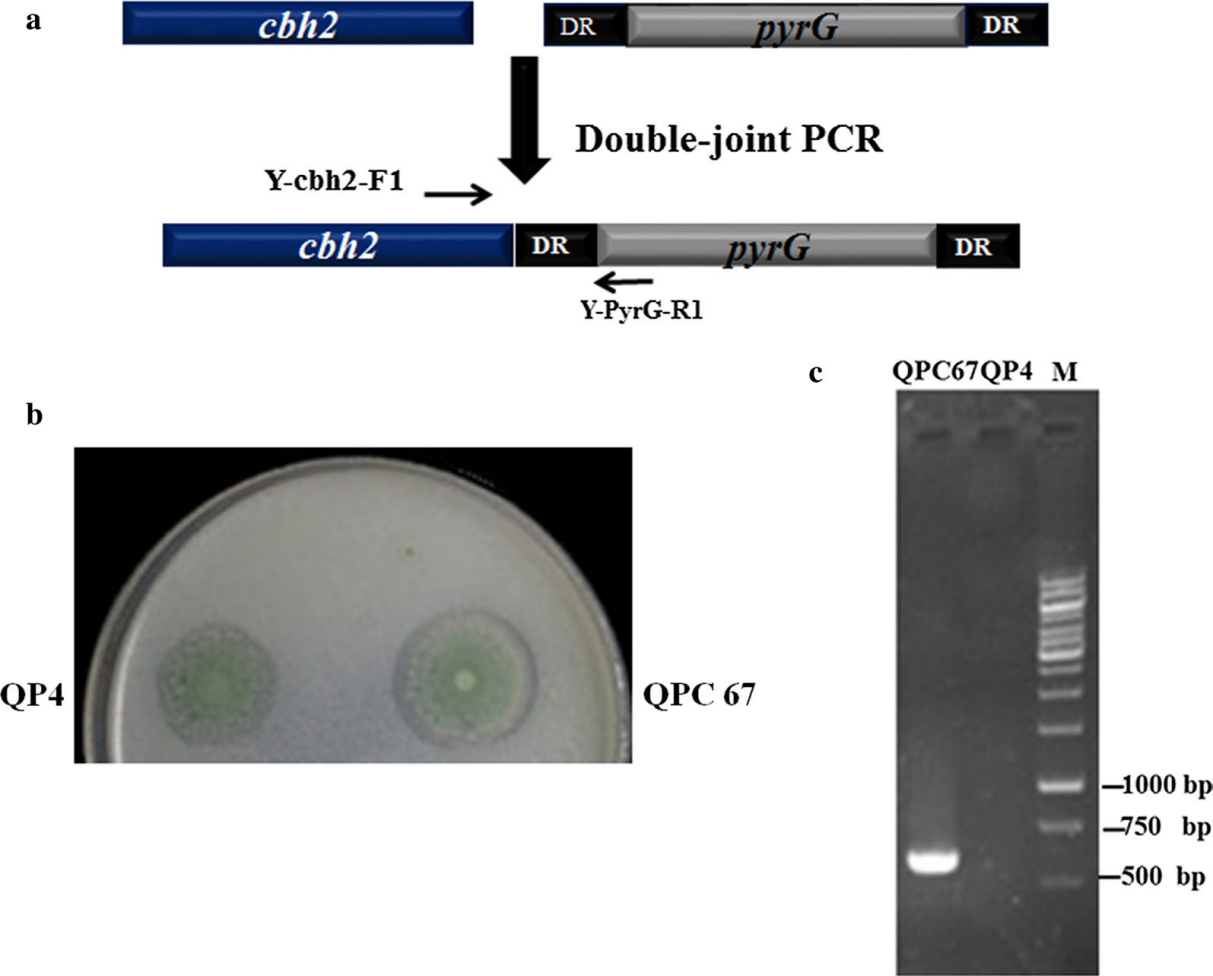
Construction of the *T. reesei cbh2* overexpression strains. **a** Cassettes used for *cbh2* overexpression in the uracil auxotrophic strain QP4. **b** The cellulose agar plate used to screen for the *cbh2*-overexpression transformants. **c** PCR confirmation of the *cbh2*-overexpression transformant QPC67, which shows a 600-bp DNA fragment product using the primers Y-cbh2-F1 and Y-PyrG-R1

The *cbh2* (Gene ID: 72567) expression cassette, *cbh2*–*pyrG*, containing the *cbh2* gene and a *pyrG* marker (*pyrG*+DR) was constructed by double-joint PCR [29] (Fig. 1a). Then the cassette was transformed into the protoplasts of the *T. reesei* uracil auxotrophic strain QP4 using the PEG-mediated method. The transformants were then screened on AMM plates containing Avicel as the sole carbon source. It was reported that growth rates of *T. reesei* CBH overexpression transformants on cellulose-containing plates correlated well with their CBH activities [16]. Here, the fastest-growing transformant QPC67 was selected from 134 candidates and further verified by PCR for the existence of the *cbh2* expression cassette in its chromosomal DNA (Fig. 1b). QPC67 gave the PCR product of 600 bp whilst here was no PCR product in the parental strain, indicating that the *cbh2* expression cassette was integrated into the genome of recombinant *T. reesei* (Fig. 1c).

**Fig. 2:**
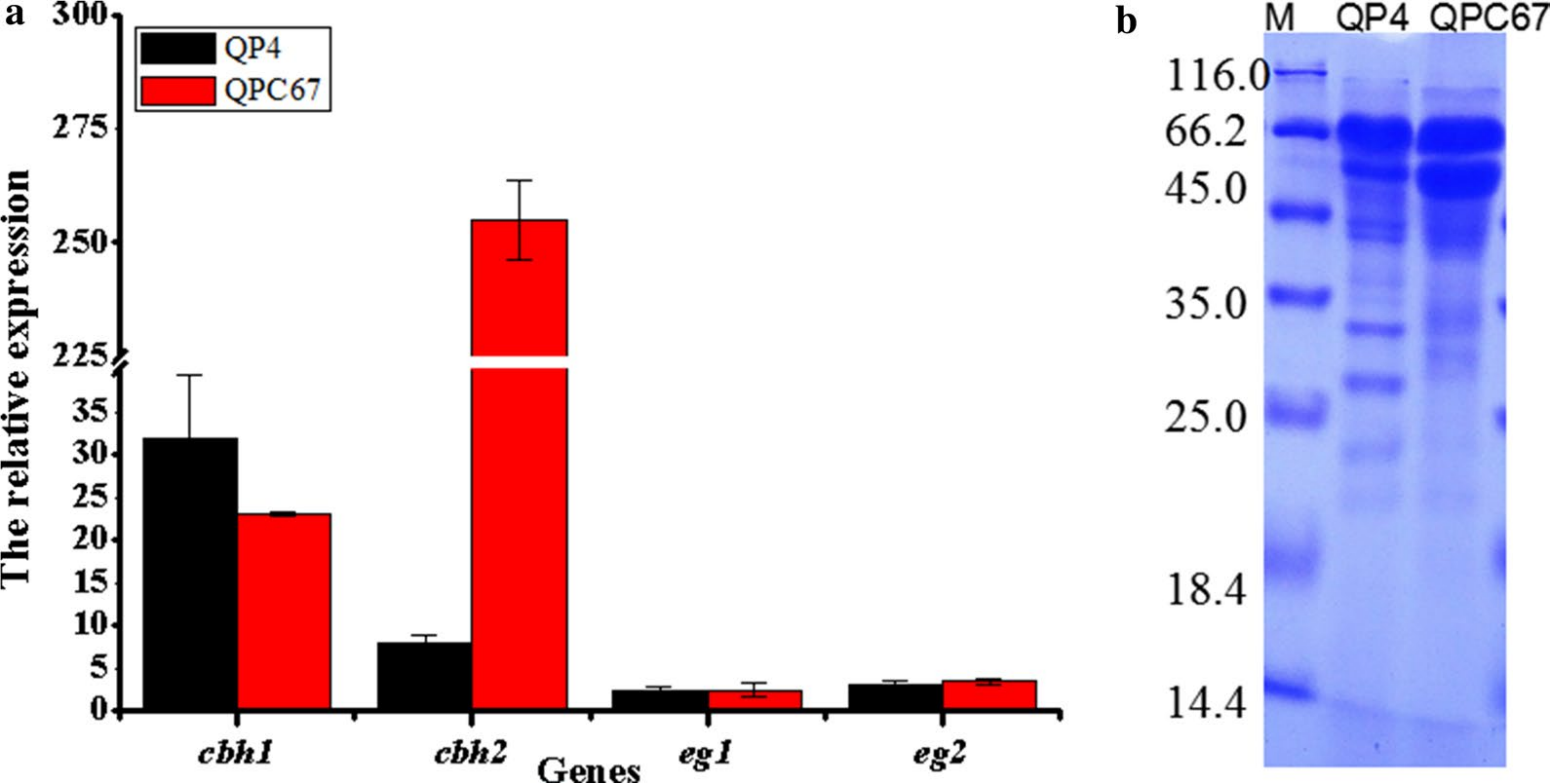
RT-qPCR and SDS-PAGE analysis for the CBH2 overexpression strain QPC67 and the parental strain QP4. **a** qPCR analysis of the transcription levels of the *cbh1, cbh2, egl1* and *egl2* genes in QPC67 and QP4. **b** SDS-PAGE analysis of the supernatants from QPC67 and QP4

Furthermore, quantitative real-time reverse transcription PCR (qPCR) was performed to investigate the transcript abundance of *cbh2* (Fig. 2a). The transcriptional level of *cbh2* in QPC67 exhibited 32-fold higher than that of the parental strain QP4. In addition, the SDS-PAGE and MS analysis confirmed that the CBH2 band in the overexpression transformant was significantly improved compared to that of QP4 (Fig. 2b). In particular, the CBH2 amount was abundant compared to that of CBH1, which is the dominant protein in the cellulolytic secretome [14]. These results demonstrated that the native *cbh2* gene in QPC67 was successfully overexpressed and the CBH2 amount in the *T. reesei* cellulase system was significantly improved.

### CBH2 overexpression does not markedly increase the total cellulase activity and saccharification ability

**Fig. 3:**
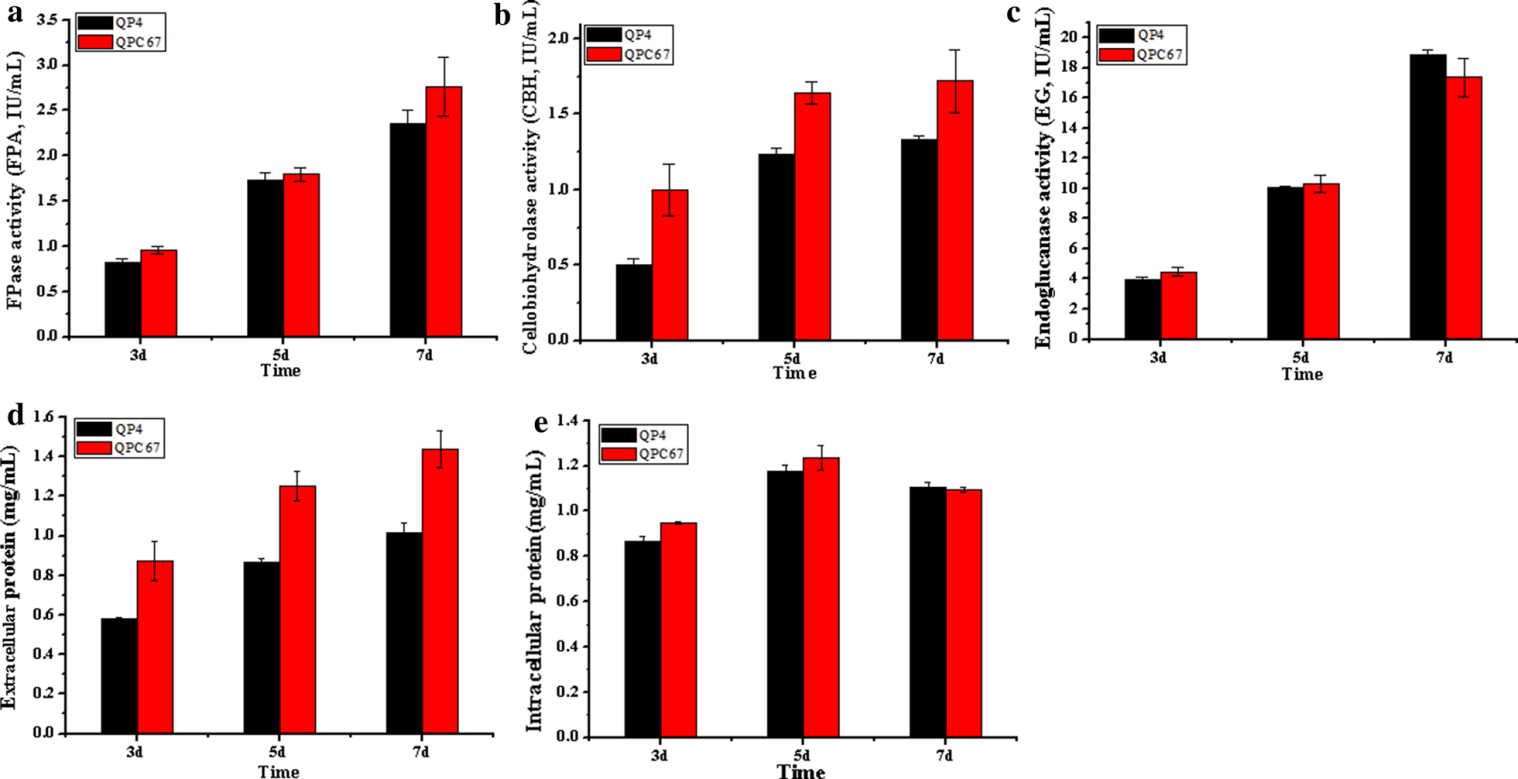
Cellulase activities and the total secreted proteins of *T. reesei* QPC67 and QP4. **a** FPase activity (FPA). **b** Cellobiohydrolase activity (CBH). **c** Endoglucanase activity (EG). **d** The total extracellular protein. **e** The total intracellular protein, which was used to determine the fungal growth. Data are means of results from three independent measurements. Error bars indicate the standard deviation

To examine the influence of CBH2 overexpression on cellulase activity, the strain QPC67 and the parent strain QP4 were cultured in cellulase-inducing medium (CM) at 30 °C for 7 days. The fermentation supernatants were collected at different time intervals. Then the activities of total cellulase (determined by filter paper assay, FPA), cellobiohydrolases, endoglucanases and the extracellular protein concentration were measured (Fig. 3). As expected, QPC67 exhibited higher cellobiohydrolase activity (over 30.0% increase) compared with that of QP4 (Fig. 3a). Accordingly, the extracellular protein secreted by QPC67 showed a 41.0% increase in comparison with that of QP4 (Fig. 3d). This could be confirmed by the above SDS-PAGE result and also correlate with the fact the cellobiohydrolases are known to account for more than 70% of the total protein secreted by *T. reesei* [30]. Correspondingly, the FPA of QPC 67 was increased by 18.0% during the late fermentation phase (Fig. 3b). However, the endoglucanase activity was not significantly changed (Fig. 3c). This was consistent with the transcription levels of the *egl1* and *egl2* gene detected by qPCR analysis (Fig. 2a). In addition, the growth rate of QPC67, which was measured by detecting the total intracellular protein, was similar to that of QP4 (Fig. 3e). These data indicated that the increase in cellulase activity and extracellular protein concentration of QPC67 was not related to fungal growth.

**Fig. 4:**
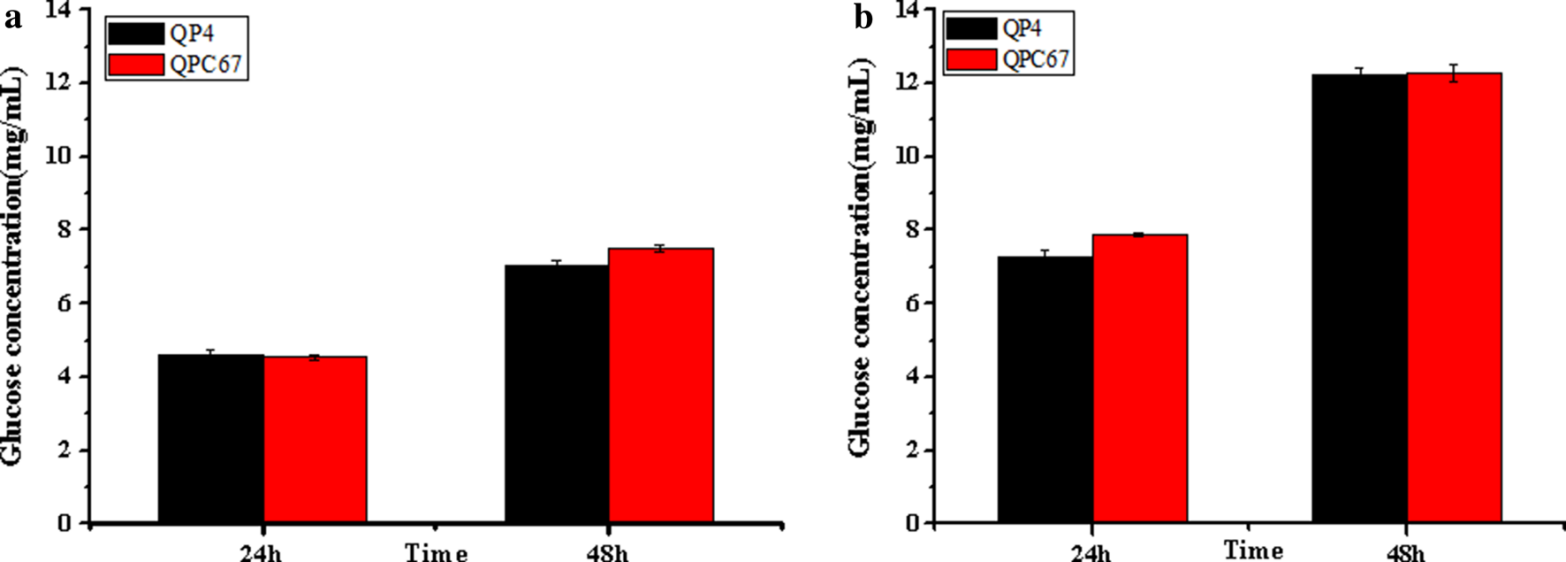
Saccharification of differently pretreated corncob residues by *T. reesei* QPC67 and QP4. **a** Saccharification of ACR with equal FPA activity. **b** Saccharification of DCR with equal protein concentration. Data are represented as the mean of three independent experiments. Error bars express the standard deviations

In order to clarify the hydrolysis ability of the cellulases produced by the CBH2 overexpression strain QPC67 on natural cellulosic materials, the crude enzyme complexes were used to saccharify differently pretreated corncob residues, acid-pretreated corncob residues (ACR) and delignified corncob residues (DCR). In the saccharification of ACR, the glucose release (7.5 mg/mL corresponding to 21.4% cellulose conversion) using the QPC67 enzyme was comparable with that of QP4 (7.1 mg/mL corresponding to 20.0% cellulose conversion) after a total enzymatic reaction of 48 h (Fig. 4a). When the DCR was used as a substrate, the final glucose yield of QPC67 (12.3 mg/mL corresponding to 33.6% cellulose conversion) was almost the same to that of QP4 (12.2 mg/mL corresponding to 33.4% cellulose conversion) (Fig. 4b). These results indicated that CBH2 overexpression could not facilitate enzymatic saccharification of differently pretreated corncob residues.

### Overexpression of the native *egl2* in *T. reesei* QP4

**Fig. 5:**
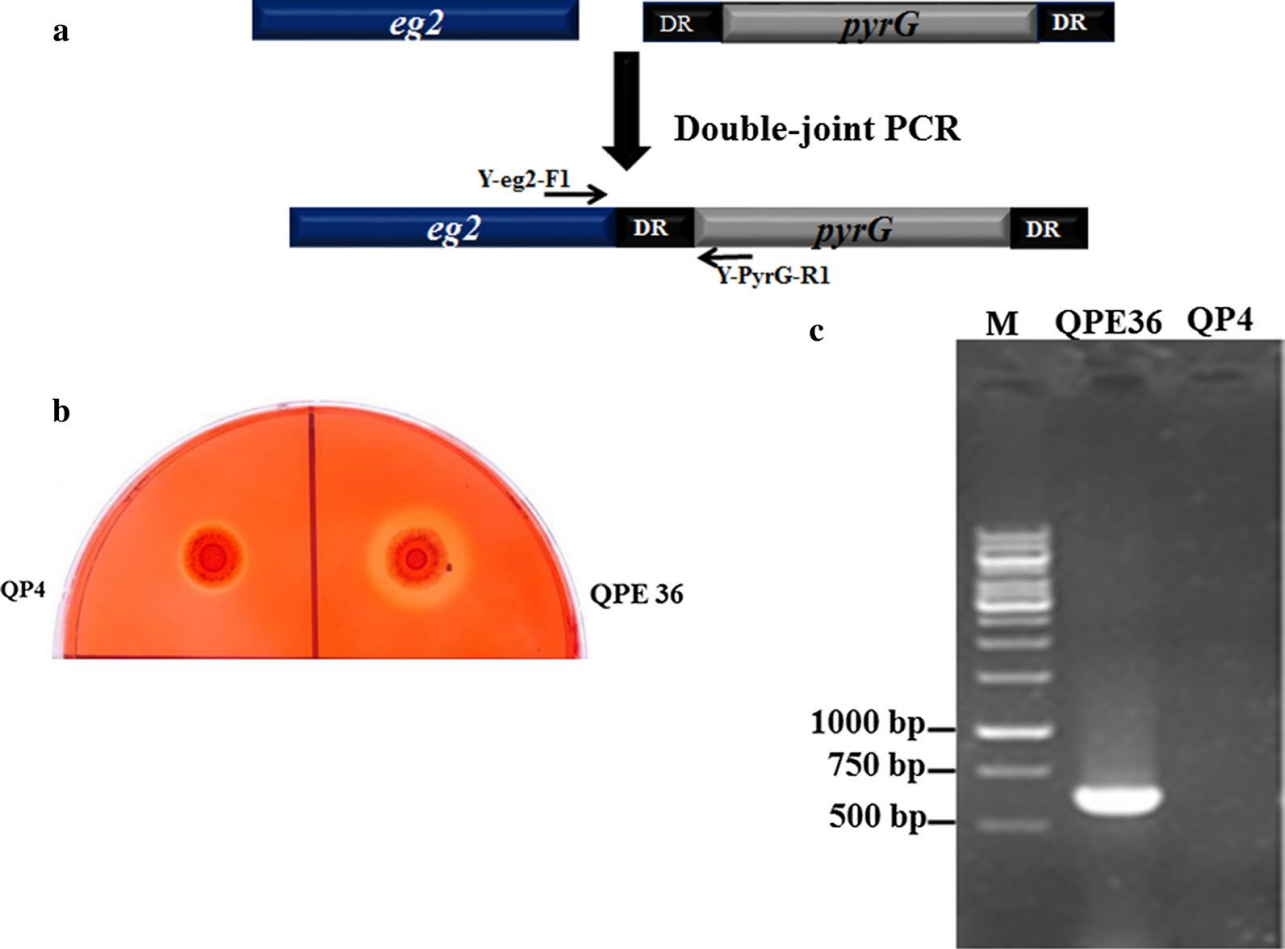
Construction of the *T. reesei egl2* overexpression strains. **a** Cassettes used for *egl2* overexpression in the uracil auxotrophic strain QP4. **b** The CMC agar plate used to screen for the *egl2* overexpression transformants. **c** PCR confirmation of the *egl2*- overexpression transformant QPE36, which shows an about 600-bp DNA fragment product using the primers Y-egl2-F1 and Y-PyrG-R1

For construction of EG2-overproducing strains, the *egl2* (Gene ID: 120312) expression cassette, *egl2*–*pyrG*, was transformed into the strain QP4 using the method as described above for CBH2 overexpression (Fig. 5a). The transformants were screened on the CMC plates containing the sodium carboxymethyl cellulose (CMC-Na) as the sole carbon source to test the endoglucanase overexpression [13]. One transformant QPE36 exhibiting the largest hydrolytic halo around the colony was selected from 128 positive *T. reesei* transformants (Fig. 5b). Then, QPE36 was verified through PCR amplification of the *egl2*–*pyrG* cassette using the genomic DNA as the template (Fig. 5c). The expected PCR product of 620 bp was obtained for QPE36 while there was no PCR product for the parental strain QP4, suggesting that the *egl2* expression cassette was inserted into the *T. reesei* chromosome.

**Fig. 6:**
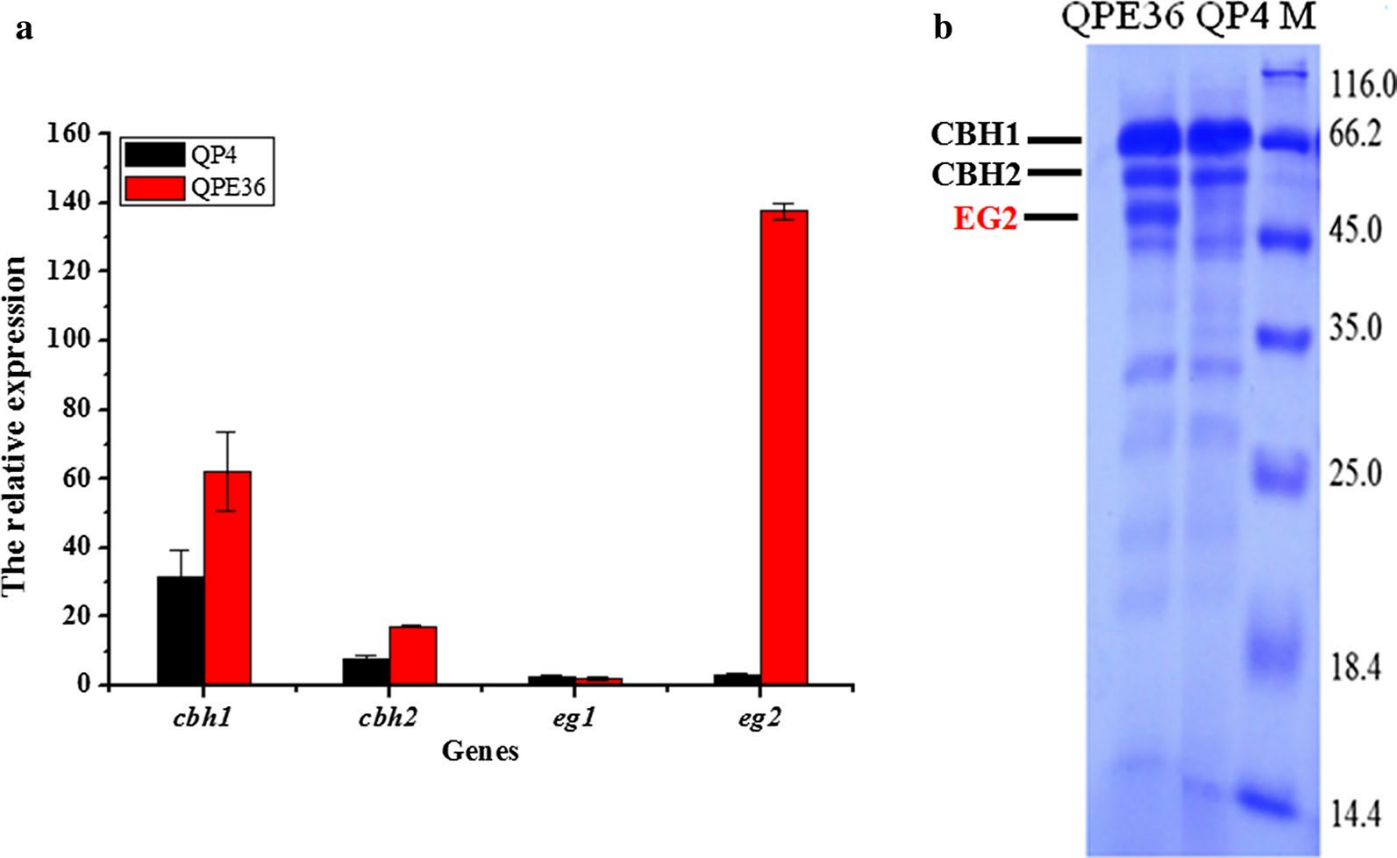
RT-qPCR and SDS-PAGE analysis for the EG2 overexpression strain QPE36 and the parental strain QP4. **a** qPCR analysis of the transcription levels of the *cbh1, cbh2, egl1* and *egl2* genes in QPE36 and QP4. **b** SDS-PAGE analysis of the supernatants from QPE36 and QP4

The transcriptional level of *egl2* in QPE36 was determined by qPCR (Fig. 6a). The result showed that the *egl2* transcript abundance in QPE36 exhibited dramatically higher (46-fold) than that of the parental strain QP4. Particularly worth mentioning is that the expression levels of cellobiohydolase genes (*cbh1* and *cbh2*) in QPE36 were also up-regulated with values of 2-fold in comparison to QP4 while the expression level of *egl1* was not affected (Fig. 6a). Moreover, the SDS-PAGE and MS analysis confirmed that the EG2 band in QPE36 was remarkably enhanced and its amount reach the level of CBH2, the protein secreted in the second largest amount after CBH1 by *T. reesei* (Fig. 6b). These results showed that the native *egl2* gene in QPE36 was successfully overexpressed and the proportion of EG2 in the *T. reesei* cellulase system was greatly improved.

### EG2 overexpression significantly enhances the total cellulase activity and saccharification ability

**Fig. 7:**
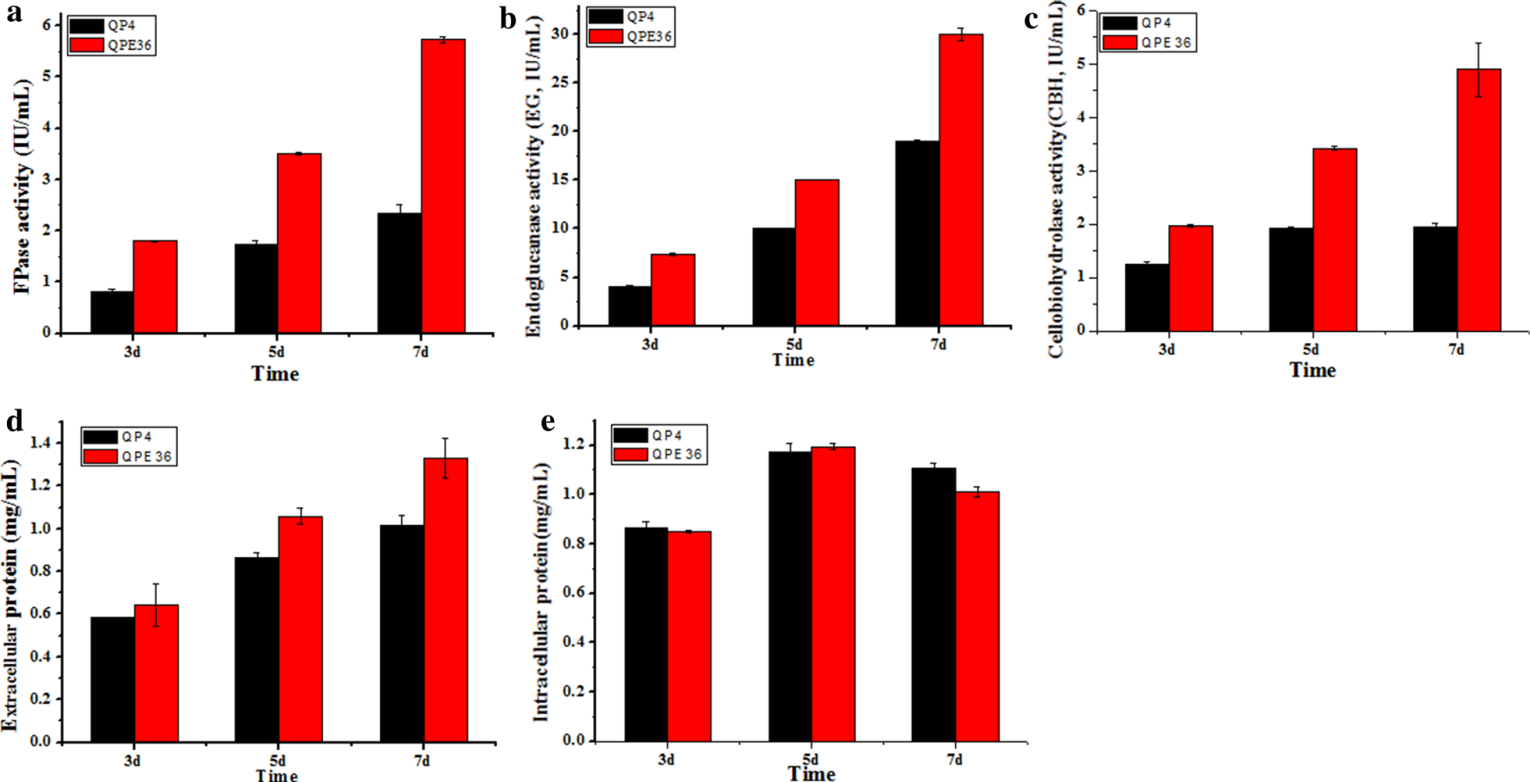
Cellulase activities and the total secreted proteins of the EG2 overexpression strain QPE36 and the parental strain QP4. **a** FPase activity (FPA). **b** Endoglucanase activity (EG). **c** Cellobiohydrolase activity (CBH). **d** The total extracellular protein. **e** The total intracellular protein, which was used to determine the fungal growth. Data are means of results from three independent measurements. Error bars indicate the standard deviation

The results of cellulase production by *T. reesei* QPE36 and QP4 were presented in Fig. 7. After 7d fermentation, the FPA of QPE36 reached 5.8 U/mL, 1.5-fold higher than that of QP4 (2.3 U/mL) (Fig. 7a). Accordingly, the amount of total secreted protein by QPE36 showed an increase of 30.6% in comparison with QP4 (Fig. 7d). To further verify the contribution of EG2 overexpression to FPA enhancement in the transformant, the activities of the major cellulase components, endoglucanases and cellobiohydrolases, were analyzed and compared between QPE36 and its parental stain QP4. As shown in Fig. 7b, c, QPE36 exhibited a 57.8% increase in the endoglucanase activity and a 1.5-fold increase in the cellobiohydrolase activity. In addition, the growth rate of QPE36 was similar to that of QP4 (Fig. 7e). These data indicated that the increase in cellulase activitiy and extracellular protein concentration of QPE36 was not related to fungal growth. Thus, overexpression of the native EG2 in *T. reesei* resulted in the remarkable increases of total cellulase activity.

**Fig. 8:**
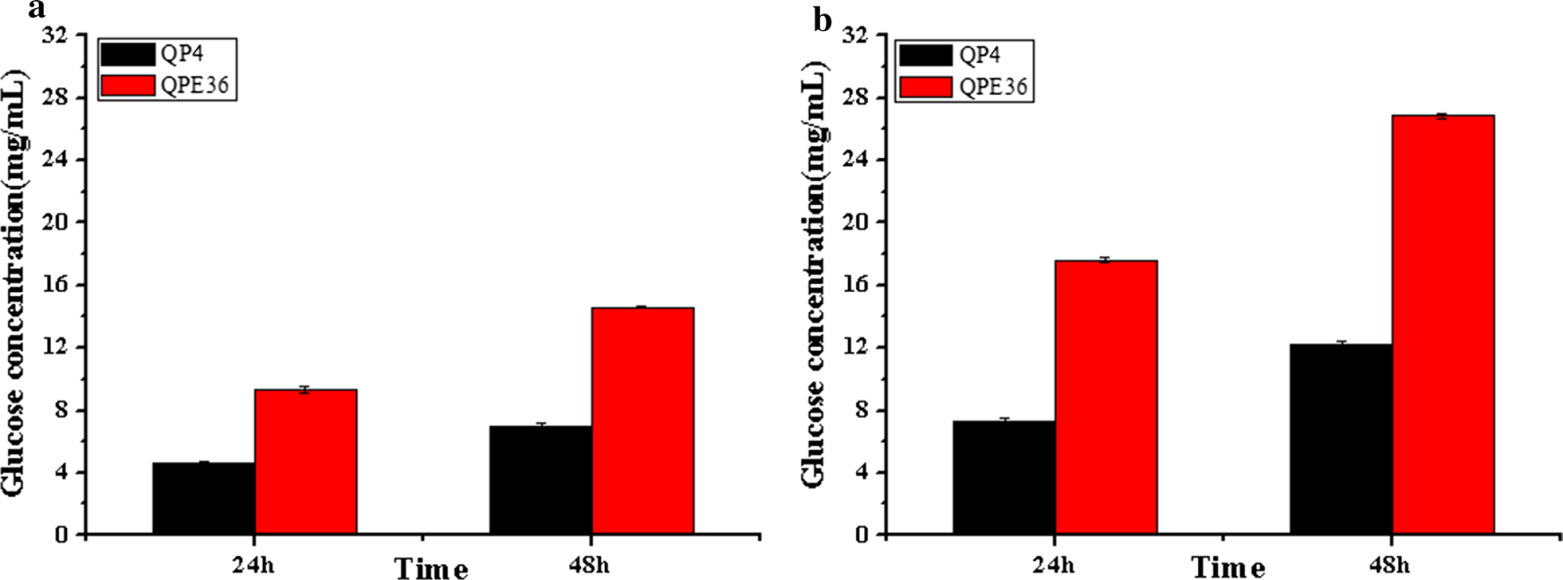
Saccharification of differently pretreated corncob residues by *T. reesei* QPE36 and QP4. Saccharification of ACR (**a**) and DCR (**b**) with same protein concentration. Data are represented as the mean of three independent experiments. Error bars express the standard deviations

Cellulase preparation derived from QPE36 was used for saccharification of the pretreated corncob residues. It was found that QPE36 released the glucose up to 14.6 mg/mL, which corresponded to 41.7% cellulose conversion, after a total enzymatic reaction of 48 h using the ACR as a substrate (Fig. 8a). As expected, the parental strain QP4 had the lower ability to hydrolyze the same substrate: only 7.1 mg/mL glucose release (that is, 20.0% cellulose conversion) was achieved after the same time reaction (Fig. 8a). When DCR was used as a substrate, the final glucose yield of QPE36 (26.9 mg/mL, corresponding to 73.4% cellulose conversion) was much higher than that of QP4 (12.3 mg/mL corresponding to 33.4% cellulose conversion) (Fig. 8b). Taken together, EG2 overexpression could facilitate construction of a more efficient cellulolytic system for optimal hydrolysis of cellulosic substrates.

### Excision of the *pyrG* marker in the EG2 overexpression strain QPE36

**Fig. 9:**
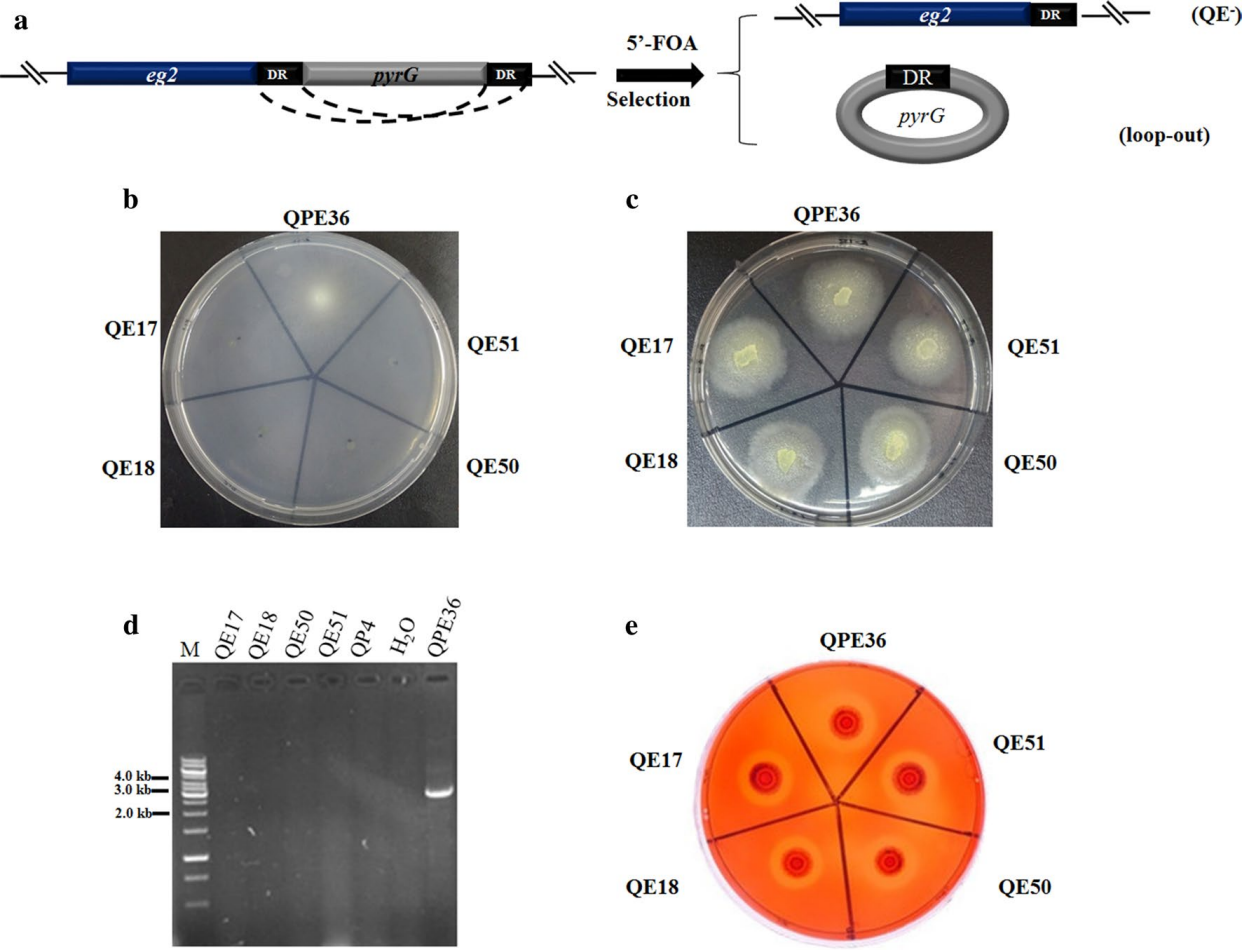
Construction of the *pyrG*-excised strains. **a** Schematic representation of the forced recombination between the DR repeat regions under 5-FOA positive selection to reobtain uracil auxotrophy. **b** Colony morphology of the 5-FOA-resistant strains grown on the MM medium without uracil. **c** Colony morphology of 5-FOA-resistant strains grown on the MM medium with uracil. **d** PCR confirmation of the absence of the *pyrG* marker in the genome of the *pyrG*-excised *T. reesei* strains. **e** The CMC plate analysis of the *pyrG*-excised *T. reesei* strains

In *T. reesei,* multiple genetic manipulations are limited by the number of readily available selection markers. Here, the bidirectionally selectable *pyrG* marker flanked by two direct repeats (DR) was used as the recyclable marker that could be excised by homologous recombination. Figure 9a shows the schematic representation of the forced recombination between the DR repeat regions under 5-FOA selection for the removal of the *pyrG* marker. To reuse the *pyrG* marker in the *egl2*-overexpression strain, the conidio spores of QPE36 was plated on 5-FOA plates. The 5-FOA resistant colonies then were transferred to minimal medium plates containing uracil or not, which allowed the growth of strains in which the marker cassette has been looped out (Fig. 9b, c). The excision frequency of *pyrG* reached 10^−3^ to 10^−4^, which was in the range reported by Hartl and Seiboth [31]. Four candidate strains, namely QE17, QE18, QE50 and QE51, were selected for further assays. PCR analysis showed that the *pyrG* gene could not be amplified from the marker-removed candidate strains while QPE36 produced an expected fragment of 2.8 kb containing the *pyrG* gene (Fig. 9d). On the same time, all four transformants could be recomplemented with the *pyrG* gene, indicating that the uridine auxotrophy was the result of the *pyrG* excision (data not shown). Moreover, the abilities of these strains to hydrolyze cellulosic substrates were evaluated on the CMC-agar plates containing uracil (Fig. 9e). All the strains showed the similar size of the hydrolytic halo around colonies to that of the parental strain QPE36, indicating that their abilities to produce cellulases were not affected after excision of the *pyrG* marker and could be used for further genetic manipulation.

### Overexpression of *bgl1* in the *pyrG*-excised strain QE51

**Fig. 10:**
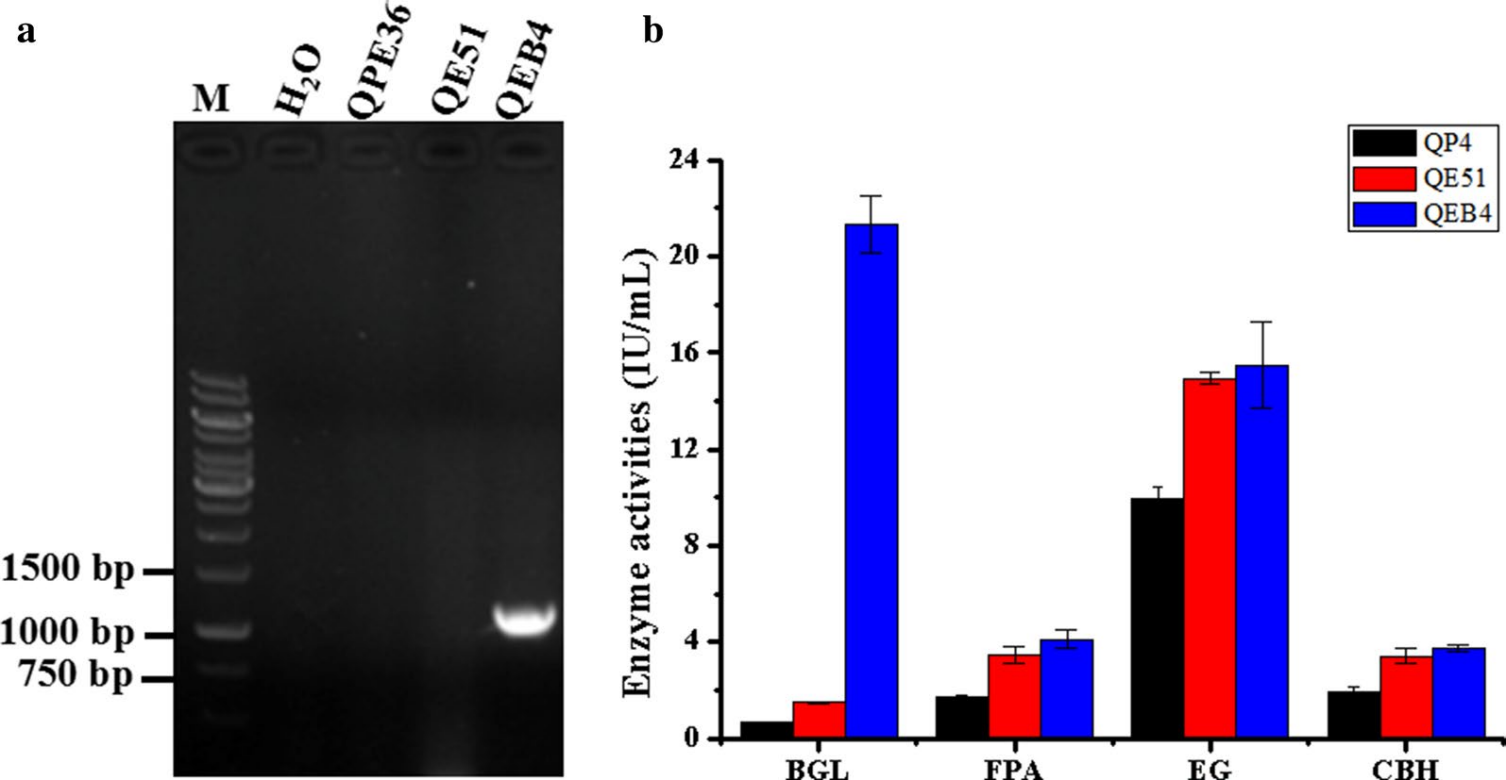
Overexpression of *bgl1* in the EG2 overexpression strain *T. reesei* QE51. **a** PCR confirmation of the BGL1 overexpression strain QEB4, which shows a 1.0-kb DNA fragment using the primers Y1 and Y2. **b** BGL, FPA, EG and CBH activities, which were measured after 5-day fermentation. Data are represented as the mean of three independent experiments and error bars express the standard deviations

It is known that BGL activity in cellulase preparations of *T. reesei* is quite low, resulting in cellobiose accumulation and thus a reduced cellulose hydrolysis efficiency [28, 32, 33]. In our previous study, the endogenous BGL1-encoding gene *bgl1* (Gene ID: 76672) was overexpressed under control of a modified *cbh1* promoter in *T. reesei*, leading to a 17.1-fold increase in BGL activity and a 65.0% increase in saccharification efficiency [26]. Here, to further improve the efficiency of the EG2-overpression cellulase system, the plasmid pTHB containing the *bgl1* overexpression cassette was co-transformed with the *pyrG*+DR fragment into *T. reesei* QE51. One transformant QEB4, which exhibited the largest black zone around the colony on the esculin plates, was selected from 140 positive transformants according to the BGL activity-screening method [24, 26]. Then, the strain QEB4 was verified through PCR amplification of the *bgl1* gene using the primers Y1 and Y2 (Fig. 10a). Furthermore, QEB4 and its ancestors, QE51 and QP4, were cultivated in CM media for cellulase production and the cellulase preparations at 5d were used for enzyme determination. It was found that QEB4 possessed the FPA, EG and CBH activities comparable to those of QE51, which were much higher than those of their original stain QP4 (Fig. 10b). As expected, QEB4 exhibited the highest BGL activity, which was 24.8 and 11.6-fold higher than those of QP4 and QE51, respectively (Fig. 10b). Consequently, the endogenous BGL1-encoding gene *bgl1* was successfully overexpressed in QE51, that is, the EG2–BGL1 double overexpression strain QEB4 was finally constructed.

### Double overexpression of *egl2* and *bgl1* results in an optimized cellulase system for saccharification of corn cob residues

**Fig. 11:**
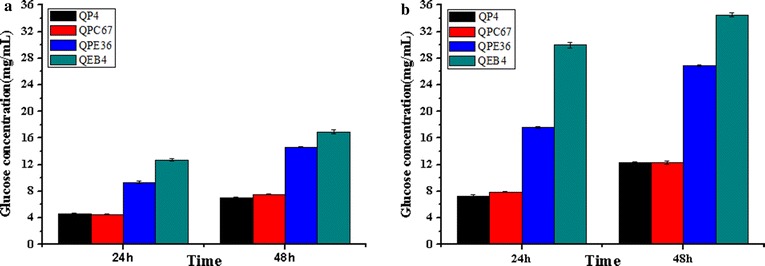
Comparison of the saccharification efficiencies towards differently pretreated corncob residues between the engineered *T. reesei* strains and the parental strain QP4. Saccharification of ACR (**a**) and DCR (**b**) by *T. reesei* QP4, QPC67, QPE36 and QEB4 with equal protein concentration. Displayed data represents averages of three independent experiments

In the above sections and the earlier reports, individual overexpression of EG2 or BGL1 could provide a remarkable hydrolysis efficiency against cellulosic biomass substrates [26, 28]. Here, the cellulase preparation from QEB4 was tested for its saccharification efficiency towards the differently pretreated corn cob residues. When the ACR were used as substrate (Fig. 11a), QEB4 exhibited much higher glucose yield after 48 h of the reaction (16.9 mg/mL, corresponding to 48.2% cellulose conversion) than the individual-overexpression strains, for which the glucose yields varied from 7.5 mg/mL (QPC67) to 14.6 mg/mL (QPE36). In hydrolysis of the DCR (Fig. 11b), QEB4 was more superior and provided nearly complete cellulose conversion (94.2%, that is, 34.5 mg/mL glucose yield) after 48 h reaction, while QPC67 and QPE36 were notably less effective, for which the cellulose conversion was 33.6 and 73.4%, respectively. These results demonstrated the EG2–BGL1 double overexpression provided a highly significant increase in saccharification efficiency compared to the individual ones, that is, combined overexpression of the major cellulase components could construct an optimized cellulase system for biomass conversion.

## Discussion

The key to the development of an economically viable lignocellulose bioconversion process is a reduction in the cost of enzymes used for depolymerization of the recalcitrant materials, which mostly depends on the improved efficiency of the cellulolytic system [24]. It is known that individual components of the system have limited hydrolytic activity while the cellulase mixture can exhibit a synergistic effect, which is closely connected with the ratios of the individual enzymes [34]. Therefore, optimization of the cellulase mixture by altering their ratios or even components has recently gained increasing recognition. These efforts involve supplement of purified cellulase components into the native cellulolytic system, construction of completely synthetic enzyme mixtures, and heterologous expression of individual enzymes in the cellulase producers [15, 35–37]. Although the cellulolytic system produced by *T. reesei* contains intact enzyme components essential to extensive hydrolysis of lignocellulose, the potential of the native system by modulating their ratios through genetic improvement strategy has not been fully exploited. In this study, multigene-expression manipulations, in combination with the use of glucose yield as a measure of the biomass conversion rate, were carried out in the hypercellulolytic fungus *T. reesei* to increase the ratios of the major endogenous cellulase components, and finally an optimized cellulase system for efficient hydrolysis of corncob residues was obtained.

Genetic manipulation of industrially important fungi has shown to be powerful tools for improving production levels, but this is largely dependent on selection marker systems [38]. Although the genetic transformation was achieved for the cellulolytic fungus *T. reesei* in 1987, only a small number of dominant and auxotrophic markers are available, restricting the multiple sequential genetic modifications in this fungus [39, 40]. To overcome this obstacle, a marker recycling system was recently established, where the auxotrophic marker *pyrG* with a direct repeat can be excised via direct-repeat homologous recombination in the presence of 5-FOA, allowing multiple rounds of gene targeting in the same strain [31, 41]. This system was successfully exemplified by the deletion of the gluco- and hexokinase encoding genes [31]. More recently, multiple protease genes were successively disrupted in *T. reesei* using the *pyrG* recyclable marker system to develop the fungus for producing therapeutic proteins [41]. However, this marker recycling system has not been applied to strain improvement of *T. reesei* for cellulase production. Here, the potential of endogenous cellulase components in *T. reesei* for enhancing cellulolytic efficiency was exploited using this strategy. Firstly, individual overexpression of the major cellulases (CBH2, EG2 or BGL1) with the *pyrG* marker was used for strain improvement to increase the cellulase production as well as the biomass conversion efficiency (Figs. 3, 7). The EG2-overexpression strain QPE36 showed significantly higher total cellulase activity and cellulolytic ability than its parental strain (Figs. 7, 8). Then, the *pyrG* marker was successfully excised by selection for resistance to 5-FOA in QPE36 (Fig. 9). The BGL1-encoding gene *bgl1* was further overexpressed in the *pyrG* excisional strain QE51 with the same *pyrG* marker to construct the EG2–BGL1 double overexpression strain QEB4, which showed much higher cellulase activity and cellulolytic ability than QE51 and QPE36. These results indicated that the *pyrG* recyclable marker system was a versatile toolkit towards efficient exploitation of the genetic resource of *T. reesei* for cellulase expression and hence biomass bioconversion.

Different types of optimized synthetic mixtures with purified component enzymes were recently designed for efficient deconstruction of lignocellulosic substrates to soluble sugars [9–11, 16–18]. For example, Billard et al. determined the optimal *T. reesei* enzyme composition for the hydrolysis of wheat straw by a statistical model, and suggested that high hydrolysis yields can be obtained from the enzyme mixture comprising CBH1 and CBH2 as the majority of the cocktail (> 50%) and also necessitate a relatively high proportion (5–10%) of EG2 [37]. These results highlighted the importance of the respective enzyme components in the development of an optimized cellulylotic system for lignocellulose bioconversion. However, the amount of CBH2 (10–15% of total protein) is much lower than that of CBH1 (50–60% of the total protein). Especially, CBH2 was shown to be the predominant cellulase component located on the conidial surface, thus probably acting as a “sensor” to effectively initiate cellulose degradation [42–44]. Therefore, this potential correlation between cellulase production and CBH2 level in *T. reesei* provided an interesting basis for genetic improvement of cellulase producers. In this study, the transcriptional level of *cbh2* in the overexpression strain QPC67 exhibited a 32-fold increase (Fig. 2a) and the amount of CBH2 secreted by QPC67 was significantly enhanced to the level of CBH1, the most abundant single protein in the cellulase mixture (Fig. 2b). However, the total cellulase activity showed an increase by only 17.0% (Fig. 3a). Recently, Fang and Xia reported that overexpressed *cbh2* in *T. reesei* via *Agrobacterium*-mediated transformation (AMT) resulted in a 4.3-fold increase in FPA, but they didn’t provide the experimental evidence of increased mRNA or protein level for CBH2 [16]. In this case, it could not exclude the possibility that this increase in FPA activity was due to the T-DNA insertion mutagenesis of AMT, since different integration loci in the fungal genomes could cause variable expression level of the target gene [45]. Here, it is also found that the saccharification abilities towards two differently pretreated corncob residues were not increased significantly for the CBH2 overexpression strain QPC67 (Fig. 3). In combination with the previous observation that high yields from hydrolysis of steam-exploded wheat straw could be obtained over a wide range of CBH1 to CBH2 ratios [37], it can be speculated that the ratio of CBH2 in the cellulolytic system is not crucial for efficient hydrolysis of certain lignocellulosic materials.

Among the endoglucanases produced by *T. reesei*, EG2 is proposed to account for most of the endoglucanase activity, because its absence reduces the endoglucanase activity by as much as 55% [23]. Furthermore, the EG2-deletion strain showed a significant drop in the total cellulase activity with lactose as the carbon source, indicating an important role of EG2 in the enzymatic hydrolysis of cellulosic substrates [23]. On the other hand, it was found that basal endoglucanase levels were present in the conidia of *T. reesei* and probably involved in the induction of cellulase synthesis by initially attacking cellulose to form oligosaccharides or cellobiose and thus finally become converted to the true inducers [46]. Indeed, on crystalline cellulose as the only carbon source, the EG2-deletion strain showed no expression of the other cellulase genes, demonstrating that EG2 is of major importance for the efficient formation of the inducer from cellulose in *T. reesei* [43]. Here, EG2 was overexpressed in the hypercellulolytic strain *T. reesei* QP4. The transcript level of *egl2* was increased by 46-fold and the secreted EG2 reached up to the protein level of CBH2, the second large amount of protein secreted by *T. reesei* (Fig. 6). Our data further exhibited that overexpression of EG2 increased the EG activity by 57.8%, the CBH activity by 170% and the total cellulase activity by 150% (Fig. 7). Thus, it can be concluded that EG2 has an important impact not only on the endoglucanase activity, but also for the total cellulase activity. This is also in agreement with the results obtained with EG2-deletion strain of *T. reesei* [43]. In addition, the cellulase preparation of the high EG2 activity-producing strain QPE36 proved to significantly improve the saccharification effect above that of its parental strain QP4 when the same enzyme dosage was used for hydrolysis of differently pretreated corncob residues (Fig. 8). That is, the same saccharification effect could be obtained with a considerably low enzyme dosage when using the EG2-overexpression cellulase preparation, thus contributing to reduction of the enzyme cost in cellulose bioconversion.

During enzymatic degradation of cellulose, BGL has the capacity to hydrolyze cellobiose to glucose in the final step and relieve the feedback inhibition of cellobiose on the activities of CBH and EG [28]. However, it is generally recognized that the insufficiency of BGL in the cellulase complex of *T. reesei* is one of the bottlenecks in efficient cellulose hydrolysis [47]. To resolve this obstacle, several strategies have been adopted to increase the ratio of BGL in the cellulase preparations, such as construction of recombinant *T. reesei* strains with high BGL activity. Nevertheless, only overexpression of BGL could not significantly increase the total cellulase activity. For example, Zhang et al. overexpressed the native *bgl1* gene in *T. reesei* RUT-C30 and obtained a strain with 5.7-fold higher BGL activity, however, the total cellulase activity was not enhanced [28]. Ma et al. reported that heterologous expression of the *bgl1* gene of *P. decumbent* in *T. reesei* RUT-C30 led to eightfold increase in BGL activity and only 30.0% increase in FPA [9]. In our previous work, the endogenous *bgl1* gene was overexpressed in *T. reesei* SP4 and a 17.1-fold increase in BGL activity was found, which resulted in only 20.0% increase in FPA [26]. When these BGL-overpression enzyme preparations were used for biomass saccharification, they exhibited remarkably higher saccharification efficiency their parental ones. Therefore, BGL overexpression could improve the saccharification ability of the cellulase system. In this study, the native BGL1 was further overexpressed in the EG2-overexpression strain QE51 and the engineered EG2–BGL1 double-overexpression strain QEB4 exhibited an over tenfold higher BGL activity than its ancestors (Fig. 10). Especially, the cellulase system produced by this strain showed a significant increase in saccharification efficiency towards differently pretreated corncob residues, for instance, a nearly complete cellulose conversion (94.2%) after 48 h enzymatic saccharification of the DCR substrate (Fig. 11).

## Conclusions

This study adopted an efficient genetic manipulation strategy based on the *pyrG* marker for overexpression of the major cellulase components in a hypercellulolytic *T. reesei* strain and explored the potential of the endogenous *T. reesei* cellulase system for biomass conversion. We observed remarkable boost in total cellulase activity and saccharification efficiency for the EG2 overexpression strain, suggesting EG2 as an enzyme component of particular importance for cellulase production and cellulolytic ability. Combining overexpression of EG2 and BGL1 provided a significantly more efficient in saccharification of pretreated corncob residues. The engineered cellulase system exhibited a nearly complete cellulose conversion after 48 h enzymatic saccharification of the DCR substrate. These results illustrate the feasibility of developing the optimized lignocellulolytic system by genetically exploiting the potentials of endogenous cellulases and suggest a prospective strategy for future improvement of industrial strains to allow the low-cost production of lignocellulose-based biofuels.

## Methods

### Fungal strains and culture conditions

*Trichoderma reesei* QP4, an uracil auxotrophic strain that was constructed from *T. reesei* QM9414 [48], was used as host strain for transformation and genomic DNA preparation. The fungal strains were cultivated on PDA plates supplemented with 0.1% (w/v) uracil when necessary at 30 °C for 5–7 days to harvest conidia. Then, the conidia were counted on hemocytometer and 10^8^ spores were transferred into 150 mL of the CM medium for enzyme production supplemented with 0.1% (w/v) uracil when necessary. The CM was composed as follows: 2% microcrystalline-cellulose, 0.5% (NH4)_2_SO_4_, 0.5% KH_2_PO_4_, 0.06% MgSO_4_·7H_2_O, 0.1% CaCl_2_·2H_2_O and 2% corn steep liquor. To assay the transcript levels of cellulase genes, 10^8^ spores were pre-cultured in 150 mL of glucose minimal medium (GMM) at 30 °C for 36 h, and subsequently 1 g of mycelia were transferred into 150 mL of Avicel minimal medium (AMM) supplemented with 0.1% (w/v) uracil when necessary. The GMM medium was composed of 2.0% glucose, 1.5% KH_2_PO_4_, 0.5% (NH4)_2_SO_4_, 0.06% MgSO_4_·7H_2_O, 0.06% CaCl_2_, 0.2% peptone, 0.001% FeSO_4_·7H_2_O, 0.00032% MnSO_4_·H_2_O, 0.00028% ZnSO_4_·7H_2_O, 0.0004% CoCl_2_, or supplemented with 0.1% (w/v) uracil when necessary. The AMM medium contained 1.0% Avicel substituted for 2.0% glucose as the sole carbon source and the remaining components in GMM.

### Construction of the CBH2 and EG2 overexpression strains

In the study, the expression cassette of *cbh2*–*pyrG* or *egl2*–*pyrG* was constructed with the double-joint PCR method [29]. The HiFi DNA Polymerase (TransGen, Beijing, China) was used for PCR amplification. All the primers were designed using the primer premier 5.0 software. DNA fragments were purified using Gel Extraction Kit (Omega, USA). Primer synthesis and DNA sequencing were performed at Sangon Inc (Shanghai, China). Oligonucleotides used in this study were listed in Table 1. The *cbh2* gene containing its own promoter and terminator regions was generated from the genomic DNA of QM9414 using the primer pair CBH2-1183UF/CBH2-2817DR. The *egl2* gene containing the native promoter and terminator regions was amplified from the genomic DNA of QM9414 using the primer pair EG2-1524UF/CBH2-1813DR. The *pyrG*+DR cassette containing the *Aspergillus niger pyrG* gene and the **d**irect **r**epeat (DR) region was constructed in three steps. Firstly, a 2.8-kb *pyrG* gene was amplified by the primer pair PyrG-S/PyrG-A used the plasmid pAB4-1 as template [49]. Secondly, a 458-bp DR fragment was generated from the 3′end of *pyrG* by PCR using the primer pair DR-S/DR-A. Thirdly, the DR fragment was fused into the 5′ end of the *pyrG* gene. Subsequently, the *cbh2* gene (or the *egl2* gene) and the *pyrG*+DR cassette were further fused together by the primer pair CBH2-1179UF/pyrG-1172DR (EG2-1524UF/pyrG-1172DR) to generate the final expression cassette, *cbh2*–*pyrG* or *egl2*–*pyrG* (Figs. 1a,  5a). The overexpression cassettes were purified and transformed into the protoplasts of *T. reesei* QP4 by the PEG-mediated transformation, which was described previously [39]. The transformants were directly screened on MM. To screen of the CBH2 overexpression strains with high cellulase activity, equivalent squares of agar piece containing the growing mycelia were further cultured on AMM agar plates. Likewise, the CMC plates, containing 1% CMC–Na (sodium carboxymethyl cellulose), 0.1% yeast extract, and 2% agar, were utilized to select the EG2 overexpression strains.

**Table 1:** Primers used in this study

Primers	Sequences (5′–3′)	Employment
CBH2-1183UF	TGAAACCCCTCACTACTGCCAT	*cbh2* overexpression strain construction
CBH2-2817DR	CTAATGCCTCGGGCTGGGACAACGAAATGGTAGGGTACGGTCAG	*cbh2* overexpression strain construction
EG2-1524UF	GACAAGAAATCGGGTGTTTAGGT	*egl2* overexpression strain construction
EG2-1813DR	CTAATGCCTCGGGCTGGGACAAATAAGAATGCGGCCGCGTGGGCTGGGTAGGGTTTG	*egl2* overexpression strain construction
PyrG-S	CTTCCTAATACCGCCTAGTCAT	*PyrG* markers amplification
PyrG-A	AGCCGCTGGTCAATGTTATC	*PyrG* markers amplification
DR-S	TTGTCCCAGCCCGAGGCATTAG	*PyrG* markers amplification
CBH2-1179UF	ACCCCTCACTACTGCCATTTAT	*cbh2* overexpression strain construction
pyrG-1172DR	TATCAATCTGGGGTAACGGACG	*cbh2/egl2* overexpression strain construction
Y-cbh2-F1	TTCACCTCCTCTTAGTGCAG	*cbh2* overexpression strain verification
Y-PyrG-R1	TACGGTCGCATAGCAGTG	*cbh2/egl2* overexpression strain verification
Y-egl2-F1	GCATATGTCAAATTTGGAGCGG	*egl2* overexpression strain verification
Y1	GCCAGGGATGCTTGAGTGTA	*bgl1* overexpression strain verification
Y2	CCCAGCCACAGGACCAAGTATG	*bgl1* overexpression strain verification
real-cbhl-Sl	GGTGGCGTGAGCAAGTATCC	Used for RT-PCR
real-cbhl-Al	TGTCCTCCAATGCCCGTGTT	Used for RT-PCR
real-cbh2-S	CTGGTCCAACGCCTTCTTCA	Used for RT-PCR
real-cbh2-A	GACCCAGACAAACGAATCCAG	Used for RT-PCR
real-actin-S	CCCAAGTCCAACCGTGAGA	Used for RT-PCR
real-actin-A	CAATGGCGTGAGGAAGAGC	Used for RT-PCR
real-egl1-S1	GGCTCGCTCTACCTGTCTCA	Used for RT-PCR
real-egl1-A1	GGGTGCCGTTCCTCCAT	Used for RT-PCR
real- egl2 -S1	ACGAGCCTTTGGTCGCAGTT	Used for RT-PCR
real- egl2-A1	GGCAGCCCAGGTGTTGATGT	Used for RT-PCR

### Re-creation of an uracil auxotrophic EG2 overexpression strain

To re-create the uracil auxotrophic strains, 10^6^ conidia of a confirmed EG2 overexpression transformant, QPE36, were spread onto the transformation plates containing 1 mg/mL uracil and 1.5 mg/mL 5-FOA, and then the cultures were incubated at 30 °C for 3 days. The candidates were further screened on minimal medium containing 1 mg/mL uracil and 1.5 mg/mL 5-FOA. The generated uracil auxotrophic strains were verified by PCR amplification of the *pyrG* gene using the primer pair pyrG-S/pyrG-A.

### Construction of the EG2 and BGL1 double overexpression strains

The plasmid pTHB [28], carrying the *T. reesei bgl1* gene expression cassette, was co-transformed with the *pyrG*+DR cassette into the protoplasts of the uracil auxotrophic EG2 overexpression strain QE51 by the PEG-mediated transformation. Esculin-plates, containing 0.3% esculin, 1% CMC–Na, 0.05% ferric citrate and 2% agar, were prepared and utilized to confirm the BGL1 overexpression strain. Then, the candidate strains were further verified by PCR using the primer pair Y1/Y2.

### RNA extraction and quantitative real-time reverse transcription PCR

For RNA extraction, 10^8^ spores were pre-cultured in minimal medium with 1% glucose at 30 °C for 36 h. The mycelia were harvested and transferred into the induction medium containing 1% cellulose at 30 °C for 20 h, then total RNA were isolated with the RNAiso™ reagent (TaKaRa, Japan). Synthesis of cDNA from total RNA was performed using PrimeScript RT reagent Kit (Takara, Japan) following the manufacturer’s description. LightCycler 480 System were used for qRT‑PCR (Roche Diagnostics, Germany). The 10 μL reaction mixtures containing 1× SYBR *Premix Ex Taq*™, 0.2 μmol/L forward primer, 0.2 μmol/L reverse primer, and 1 ul cDNA template (tenfold diluted) using the SYBR *Premix Ex Taq*™ (Tli RNaseH Plus) kit (Tkara, Japan) were performed in triplicated. qRT‑PCR protocols were as following: initial denaturation of 1 min at 95 °C, followed by 40 cycles of 5 s at 95 °C, 20 s at 60 °C. Melting curve analysis from 65 to 95 °C was performed to confirm the specific of the applications. LightCycler480 software 1.5.0 was used for calculate Ct value. Transcript levels of target genes were normalized against the level of actin gene with ddCt method [50].

### Cellulase activity assay, protein measurement and SDS-PAGE assay

The total cellulase activity (Filter paper activity, FPA) was measured using Whatman No. 1 filter paper as substrate. The reaction mixtures contained 50 mg of filter paper, 1.5 mL of 50 mM citrate buffer (pH 4.8), and 500 µL of the suitably diluted enzyme fractions. These mixtures were then incubated at 50 °C for 60 min. EG activities were assayed with CMC–Na as substrate. The enzyme reactions were performed in 2 mL of 1% substrate citrate buffer (pH 4.8) at 50 °C for 30 min. The amount of reducing sugar released was determined using the DNS method [51]. Cellobiohydrolase (CBH) activity was assayed as reported by Fang and Xia [16]. One unit of FPA, the EG activity or the CBH activity was defined as the amount of enzyme to liberate one micromole (μM) reducing sugars per minute. The β-glucosidase (BGL) activity was determined according to Ghose with modifications using p-nitrophenyl-β-d-glucopyranoside (pNPG) as a substrate [51]. The diluted supernatants (100 μL) were incubated with 50 μL of 10 mM pNPG dissolved in 50 mM acetate buffer (pH 5.0) at 50 °C for 30 min. Then, 150 μL of each sample was mixed with an equal volume of 10% sodium carbonate. The absorbance at 420 nm was measured. One unit of BGL activity was defined as the amount of enzyme releasing 1 μmol of pNP per minute. Since it is difficult to separate the mycelial biomass from the insoluble cellulose substrate in the cellulase production medium, growth rates of *T. reesei* strains were measured by detecting the total intracellular protein amount extracted by 1 M NaOH [52]. Protein concentration of each culture supernatant was determined using a Bio-Rad DC Protein Assay kit (Sangon Biotech, Shanghai, China) including bovine serum albumin standards. Three biological triplicates were designed in all experiments. SDS-PAGE electrophoresis was performed in 12% polyacrylamide separating gel.

### Saccharification of the pretreated corncob residues

The corncob residues were kindly provided by LONGLIVE Co., Yucheng, Shandong province, China. Acid-pretreated (ACR) and delignified (DCR) corncob residues were used as substrates in the saccharification process and the components of these substrates had been described by Liu et al. [53]. The cellulase crude complexes for the saccharification of the pretreated corncob residues were placed in 100 mL flasks containing 30 mL reagent using 5% (w/v) of corncob residues as substrate. Enzyme loading was 2.5 mg protein/g substrate. The pH value and temperature were adjusted to 4.8 (with 50 mM citric acid buffer) and 50 °C, respectively. Glucose production was detected with an SBA-40C biological sensor analyzer (BISAS, Shandong, China) after incubation for 24 or 48 h.

## Abbreviations

CBH: cellobiohydrolase
EG: endoglucanase
BGL: β-glucosidase
CMC: carboxymethyl cellulose
FPase: filter paper enzyme
*p*NPC: 4-nitrophenyl-β-d-cellobioside
*p*NPG: 4-nitrophenyl-β-d-glucopyranoside
SDS-PAGE: sodium dodecyl sulfate–polyacrylamide gel electrophoresis
ACR: corncob residues after hemicellulose extraction
DCR: alkali delignified corncob residues

## References

[1] Somerville C (2015). Next generation biofuels. AIP Conf Proc.

[2] Limayem A, Ricke SC (2012). Lignocellulosic biomass for bioethanol production: current perspectives, potential issues and future prospects. Prog Energy Combust Sci..

[3] Himmel ME, Ding S-Y, Johnson DK, Adney WS, Nimlos MR, Brady JW, Foust TD (2007). Biomass recalcitrance: engineering plants and enzymes for biofuels production. Science.

[4] Gusakov AV, Salanovich TN, Antonov AI, Ustinov BB, Okunev ON, Burlingame R, Emalfarb M, Baez M, Sinitsyn AP (2007). Design of highly efficient cellulase mixtures for enzymatic hydrolysis of cellulose. Biotechnol Bioeng.

[5] Jørgensen H, Kristensen JB, Felby C (2007). Enzymatic conversion of lignocellulose into fermentable sugars: challenges and opportunities. Biofpr.

[6] Banerjee G, Car S, Scott-Craig JS, Borrusch MS, Bongers M, Walton JD (2010). Synthetic multi-component enzyme mixtures for deconstruction of lignocellulosic biomass. Bioresour Technol.

[7] Gao D, Chundawat SPS, Krishnan C (2010). Mixture optimization of six core glycosyl hydrolases for maximizing saccharification of ammonia fiber expansion (AFEX) pretreated corn stover. Bioresour Technol.

[8] Montenecourt BS, Eveleigh DE (1979). Selective screening methods for the isolation of high yielding cellulase mutants of *Trichoderma reesei*. Hydrolysis Cellulose.

[9] Ma L, Zhang J, Zou G, Wang C, Zhou Z (2011). Improvement of cellulase activity in *Trichoderma reesei* by heterologous expression of a beta-glucosidase gene from Penicillium decumbens. Enzyme Microb Technol.

[10] Cavaco-Paulo A, Cortez J, Almeida L (1997). The effect of cellulase treatment in textile washing processes. J Soc Dyers Col.

[11] Boisset C, Fraschini C, Schülein M (2000). Imaging the enzymatic digestion of bacterial cellulose ribbons reveals the endo character of the cellobiohydrolase Cel6A from *Humicola insolens* and its mode of synergy with cellobiohydrolase Cel7A. Appl Environ Microbiol.

[12] Zhou J, Wang Y, Chu J, Luo L, Zhuang Y, Zhang S (2009). Optimization of cellulase mixture for efficient hydrolysis of steam-exploded corn stover by statistically designed experiments. Bioresour Technol.

[13] Liu G, Qin Y, Li Z (2013). Development of highly efficient, low-cost lignocellulolytic enzyme systems in the post-genomic era. Biotechnol Adv.

[14] Rosgaard L, Pedersen S, Langston J, Akerhielm D, Cherry JR, Meyer AS (2007). Evaluation of minimal *Trichoderma reesei* cellulase mixtures on differently pretreated barley straw substrates. Biotechnol Prog.

[15] Banerjee G, Car S, Scott-Craig JS, Borrusch MS, Walton JD (2010). Rapid optimization of enzyme mixtures for deconstruction of diverse pretreatment/biomass feedstock combinations. Biotechnol Biofuels.

[16] Fang H, Xia L (2013). High activity cellulase production by recombinant *Trichoderma reesei* ZU-02 with the enhanced cellobiohydrolase production. Bioresour Technol.

[17] Kubicek CP, Mikus M, Schuster A (2009). Metabolic engineering strategies for the improvement of cellulase production by *Hypocrea jecorina*. Biotechnol Biofuels.

[18] Peterson R, Nevalainen H (2012). Trichoderma reesei RUT-C30–thirty years of strain improvement. Microbiology.

[19] Bischof RH, Ramoni J, Seiboth B (2016). Cellulases and beyond: the first 70 years of the enzyme producer *Trichoderma reesei*. Microb Cell Fact.

[20] Zhou J, Wang Y, Chu J, Zhuang Y, Zhang S, Yin P (2008). Identification and purification of the main components of cellulases from a mutant strain of *Trichoderma viride* T 100-14. Bioresour Technol.

[21] Miettinen-Oinonen A, Suominen P (2002). Enhanced production of *Trichoderma reesei* endoglucanases and use of the new cellulase preparations in producing the stonewashed effect on denim fabric. Appl Environ Microbiol.

[22] Kyriacou A, MacKenzie CR, Neufeld RJ (1987). Detection and characterization of the specific and nonspecific endoglucanases of *Trichoderma reesei*: evidence demonstrating endoglucanase activity by cellobiohydrolase II. Enzyme Microb Technol.

[23] Suominen PL, Mäntylä AL, Karhunen T, Hakola S, Nevalainen H (1993). High frequency one-step gene replacement in *Trichoderma reesei*. II. Effects of deletions of individual cellulase genes. Mol Gen Genet.

[24] Berlin A, Maximenko V, Gilkes N, Saddler J (2007). Optimization of enzyme complexes for lignocellulose hydrolysis. Biotechnol Bioeng.

[25] Pryor SW, Nahar N (2015). β-glucosidase supplementation during biomass hydrolysis: how low can we go?. Biomass Bioenergy.

[26] Qian Y, Zhong L, Hou Y, Qu Y, Zhong Y (2016). Characterization and strain improvement of a hypercellulytic variant, *Trichoderma reesei* SN1, by Genetic engineering for optimized cellulase production in biomass conversion improvement. Front Microbiol..

[27] Wang B, Xia L (2011). High efficient expression of cellobiase gene from *Aspergillus niger* in the cells of *Trichoderma reesei*. Bioresour Technol.

[28] Zhang J, Zhong Y, Zhao X, Wang T (2010). Development of the cellulolytic fungus Trichoderma reesei strain with enhanced beta-glucosidase and filter paper activity using strong artificial cellobiohydrolase 1 promoter. Bioresour Technol.

[29] Yu JH, Hamari Z, Han KH, Seo JA, Reyes-Domínguez Y, Scazzocchio C (2004). Double-joint PCR: a PCR-based molecular tool for gene manipulations in filamentous fungi. Fungal Genet Biol.

[30] Vinzant TB, Adney WS, Decker SR, Baker JO, Kinter MT, Sherman NE, Fox JW, Himmel ME (2001). Fingerprinting *Trichoderma reesei* hydrolases in a commercial cellulase preparation. Appl Biochem Biotechnol.

[31] Hartl L, Seiboth B (2005). Sequential gene deletions in *Hypocrea jecorin*a using a single blaster cassette. Curr Genet.

[32] Lynd LR, Weimer PJ, van Zyl WH, Pretorius IS (2002). Microbial cellulose utilization: fundamentals and biotechnology. Microbiol Mol Biol Rev.

[33] Singhania RR, Patel AK, Sukumaran RK, Larroche C, Pandey A (2013). Role and significance of beta-glucosidases in the hydrolysis of cellulose for bioethanol production. Bioresour Technol.

[34] Lee S, Lee YH, Park JM, Bai DH, Jang JK, Park YS (2014). Bioconversion of ginsenosides from red ginseng extract using candida allociferrii JNO301 isolated from Meju. Mycobiology.

[35] Selig MJ, Knoshaug EP, Adney WS, Himmel ME, Decker SR (2008). Synergistic enhancement of cellobiohydrolase performance on pretreated corn stover by addition of xylanase and esterase activities. Bioresour Technol.

[36] Ye Z, Zheng Y, Li B, Borrusch MS, Storms R, Walton JD (2014). Enhancement of synthetic Trichoderma-based enzyme mixtures for biomass conversion with an alternative family 5 glycosyl hydrolase from Sporotrichum thermophile. PLoS ONE.

[37] Billard H, Faraj A, Ferreira NL, Menir S, Heiss-Blanquet S (2012). Optimization of a synthetic mixture composed of major *Trichoderma reesei* enzymes for the hydrolysis of steam-exploded wheat straw. Biotechnol Biofuels.

[38] Meyer V (2008). Genetic engineering of filamentous fungi—progress, obstacles and future trends. Biotechnol Adv.

[39] Penttilä M, Nevalainen H, Rättö M, Salminen E, Knowles J (1987). A versatile transformation system for the cellulolytic filamentous fungus *Trichoderma reesei*. Gene.

[40] Seidl V, Seiboth B (2010). *Trichoderma reesei*: genetic approaches to improving strain efficiency. Biofuels.

[41] Landowski CP, Huuskonen A, Wahl R, Westerholm-Parvinen A, Kanerva A, Hänninen AL, Salovuori N, Penttilä M, Natunen J, Ostermeier C (2015). Enabling low cost biopharmaceuticals: a systematic approach to delete proteases from a well-known protein production host *Trichoderma reesei*. PLoS ONE.

[42] Messner R, Kubicek CP (1991). Carbon Source Control of Cellobiohydrolase I and II Formation by *Trichoderma reesei*. Appl Environ Microbiol.

[43] Seiboth B, Hakola S, Mach RL, Suominen PL, Kubicek CP (1997). Role of four major cellulases in triggering of cellulase gene expression by cellulose in *Trichoderma reesei*. J Bacteriol.

[44] Kubicek-Pranz EM, Gruber F, Kubicek CP (1991). Transformation of *Trichoderma reesei* with the cellobiohydrolase II gene as a means for obtaining strains with increased cellulase production and specific activity. J Biotechnol.

[45] Verdoes JC, van Diepeningen AD, Punt PJ, Debets AJ, Stouthamer AH, van den Hondel CA (1994). Evaluation of molecular and genetic approaches to generate glucoamylase overproducing strains of *Aspergillus niger*. J Biotechnol.

[46] Kubicek CP (1987). Involvement of a conidial endoglucanase and a plasma-membrane-bound beta-glucosidase in the induction of endoglucanase synthesis by cellulose in *Trichoderma reesei*. J Gen Microbiol.

[47] Saloheimo M, Kuja-Panula J, Ylösmäki E, Ward M, Penttilä M (2002). Enzymatic properties and intracellular localization of the novel *Trichoderma reesei* β-glucosidase BGLII (Cel1A). Appl Environ Microbiol.

[48] Zhong L, Qian Y, Dai M, Zhong Y (2016). Improvement of uracil auxotrophic transformation system in *Trichoderma reesei* QM9414 and overexpression of β-glucosidase. CIESC J..

[49] Mattern IE, Punt PJ, Van den Hondel C (1988). A vector for *Aspergillus* transformation conferring phleomycin resistance. Fung Gene.

[50] Livak KJ, Schmittgen TD (2001). Analysis of relative gene expression data using real-time quantitative PCR and the 2^−ΔΔCT^ method. Methods.

[51] Ghose TK (1987). Measurement of cellulase activities. Pure Appl Chem.

[52] Aro N, Ilmén M, Saloheimo A, Penttilä M (2003). ACEI of *Trichoderma reesei* is a repressor of cellulase and xylanase expression. Appl Environ Microbiol.

[53] Liu K, Lin X, Yue J, Li X, Fang X, Zhu M, Lin J, Qu Y, Xiao L (2010). High concentration ethanol production from corncob residues by fed-batch strategy. Bioresour Technol.

